# *Lactobacillus reuteri* Prevents Diet-Induced Obesity, but not Atherosclerosis, in a Strain Dependent Fashion in *Apoe−/−* Mice

**DOI:** 10.1371/journal.pone.0046837

**Published:** 2012-10-09

**Authors:** Frida Fåk, Fredrik Bäckhed

**Affiliations:** 1 Sahlgrenska Center for Cardiovascular and Metabolic Research/Wallenberg Laboratory, University of Gothenburg, Gothenburg, Sweden; 2 Department of Molecular and Clinical Medicine, University of Gothenburg, Gothenburg, Sweden; Columbia University, United States of America

## Abstract

**Objective:**

To investigate whether the specific strains of *Lactobacillus reuteri* modulates the metabolic syndrome in *Apoe−/−* mice.

**Methods:**

8 week-old *Apoe−/−* mice were subdivided into four groups who received either *L. reuteri* ATCC PTA 4659 (ATCC), DSM 17938 (DSM), L6798, or no bacterial supplement in the drinking water for 12 weeks. The mice were fed a high-fat Western diet with 0.2% cholesterol and body weights were monitored weekly. At the end of the study, oral glucose and insulin tolerance tests were conducted. In addition, adipose and liver weights were recorded along with analyses of mRNA expression of ileal Angiopoietin-like protein 4 (*Angptl4*), the macrophage marker F4/80 encoded by the gene *Emr1* and liver Acetyl-CoA carboxylase 1 (*Acc1*), Fatty acid synthase (*Fas*) and Carnitine palmitoyltransferase 1a (*Cpt1a*). Atherosclerosis was assessed in the aortic root region of the heart.

**Results and Conclusions:**

Mice receiving *L. reuteri* ATCC gained significantly less body weight than the control mice, whereas the L6798 mice gained significantly more. Adipose and liver weights were also reduced in the ATCC group. Serum insulin levels were lower in the ATCC group, but no significant effects were observed in the glucose or insulin tolerance tests. Lipogenic genes in the liver were not altered by any of the bacterial treatments, however, increased expression of *Cpt1a* was found in the ATCC group, indicating increased β-oxidation. Correspondingly, the liver trended towards having lower fat content. There were no effects on inflammatory markers, blood cholesterol or atherosclerosis. In conclusion, the probiotic *L. reuteri* strain ATCC PTA 4659 partly prevented diet-induced obesity, possibly via a previously unknown mechanism of inducing liver expression of *Cpt1a*.

## Introduction

The adult human intestine is home to an almost inconceivable number of microorganisms. The size of the population–up to 100 trillion–far exceeds that of all other microbial communities associated with the body’s surfaces and is ∼10 times greater than the total number of our somatic and germ cells [1]. Accordingly, our gut microbiota can be viewed as a microbial organ placed within a host organ and is composed of different cell lineages with specific metabolic functions and with capacity to communicate with one another and the host [2]. A number of recent observations have demonstrated that the gut microbiota is altered in obese humans and that the gut microbiota may be considered an environmental factor that modulates obesity.

High-fat feeding significantly alters the gut microbial composition, with decreased levels ofBacteroidetes and an increase levels ofFirmicutes and Proteobacteria [3], [4]. Furthermore, high-fat feeding reduces bifidobacteria levels, bacteria which are known to have many physiologically positive effects, including improved mucosal barrier function. Mice fed a high-fat diet supplemented with oligofructose restored bifidobacteria levels and decreased endotoxemia, which is associated with obesity [5]. These observations suggest that increased levels of bifidobacteria may decrease intestinal permeability and lower the circulating levels of endotoxin. Furthermore, the increase in bifidobacteria correlated with improved glucose tolerance, glucose-induced insulin secretion, lower body-weight gain, and decreased production of inflammatory mediators [5].

Recently, probiotic bacteria have been tested for their ability to affect obesity. The probiotic *Lactobacillus gasseri* SBT2055 was able to reduce adiposity and body weight in obese adults consuming a fermented milk with the bacterium for 12 weeks, potentially by reducing lipid absorption and inflammatory status [6]. Another study investigated the effect of perinatal *Lactobacillus rhamnosus* GG on childhood growth patterns [7]. The probiotic modulated the body weight increase in the early years of life, but had no effect in later stages of development [7]. A recent study demonstrated that *Lactobacillus paracasei* ssp paracasei F19 (F19) prevented diet-induced obesity in mice [8].

Atherosclerosis is a chronic inflammation in the blood vessels, resulting in the build-up of fatty streaks, and with time, atherosclerotic plaques. Obesity, insulin resistance and high blood pressure are established risk factors for the disease, but recently, the gut microbiota has also been suggested to play a significant role through its processing of phosphatidylcholine in the diet, leading to pro-atherogenic metabolites in the liver [9]. Bacteria may also influence other mechanisms of atherogenesis, e.g. lactobacilli have been shown to reduce blood cholesterol levels in both rodents and humans [10], [11], potentially by modulating cholesterol re–absorption from the gut through its effects on bile acid metabolism. Only a few studies have investigated interventions with bacteria on atherosclerosis development in animal models. Portugal LR *et al* (2006 [12]) tested *Lactobacillus delbruecki* in the *Apoe−/−* mouse model, but observed no significant effects on lesion size. However, the studied bacterium showed no effects on blood cholesterol levels and the mice were colonized with *L. delbruecki* between 4 and 10 weeks of age, which can be considered relatively early in disease progression.

Here we investigated whether different strains from a well-characterized probiotic bacterial species, *Lactobacillus reuteri*, modulated metabolic phenotypes such as obesity, insulin resistance, and atherosclerosis in *Apoe−/−* mice.

**Table 1:** Primer sequences for RT-PCR quantification of mRNA expression.

*Acc1*	Forward	AAGTCCTTGGTCGGGAAGTATACA
	Reverse	ACTCCCTCAAAGTCATCACAAACA
*Angptl4*	Forward	GGAAAAGATGCACCCTTCAA
	Reverse	AATGAGCTGGGTCATCTTGG
*Cpt1a*	Forward	GCACTGCAGCTCGCACATTACAA
	Reverse	CTCAGACAGTACCTCCTTCAGGAAA
*Emr1*	Forward	TGACAACCAGACGGCTTGTG
	Reverse	GCAGGCGAGGAAAAGATAGTGT
*Fas*	Forward	TGGTGAATTGTCTCCGAAAAGA
	Reverse	CACGTTCATCACGAGGTCATG

## Materials and Methods

### Animals and Colonization

*Apoe−/−* mice were distributed into four groups at 8 weeks of age and fed a Western diet supplemented with 0.2% cholesterol (TD88137, Harlan Laboratories, Madison, USA) for 12 weeks and maintained on a 12∶12 hour light:dark cycle. *L. reuteri* ATCC 4659 (ATCC), *L. reuteri* DSM (DSM) or *L. reuteri* L6798 (L6798) were grown in MRS broth, frozen in PBS as 1 ml aliquots and administered daily, between 8 and 20 weeks of age, to the drinking water at a dose of 10^9^ ****CFU/mouse per day. Ten mice without bacterial treatment served as controls. The mice were weighed once a week and food and water consumption were measured throughout the study. At 18 and 19 weeks of age, insulin and glucose tolerance tests were conducted, and the mice were thereafter anesthetized using isofluoran inhalation and euthanized at 20 weeks of age after a 4 hour fast. After blood sampling through *vena cava*, the mice were perfused with PBS through the heart, followed by organ harvest. Aorta, heart, liver, epididymal white adipose tissue (WAT), pancreas, small intestine and cecum were saved for further analyses.

**Figure 1:**
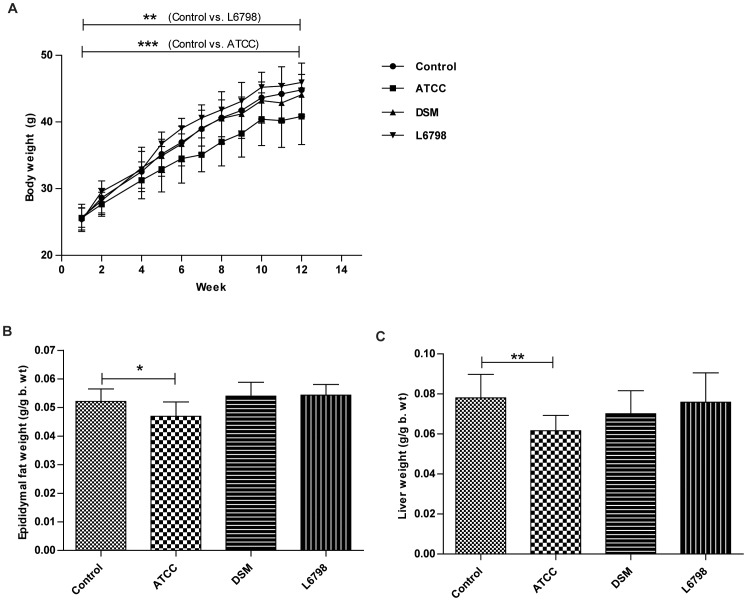
*L. reuteri* ATCC 4659 protects against diet-induced obesity in mice. Body weight gain (A), epididymal (B) and liver (C) weights in *Apoe−/−* mice treated with either *L. reuteri* ATCC 4659 (ATCC), *L. reuteri* DSM 1798 (DSM) or *L. reuteri* L6798 (L6978) in the drinking water for 12 weeks. Data shown are mean (SD). Repeated measures ANOVA was used to determine significant differences in body weight development between groups (*P*<0.0001 Control vs ATCC, *P*<0.01 Control vs L6798).

The University of Gothenburg Animal Studies Committee approved the study (ethical approval number: 15-2010).

**Figure 2:**
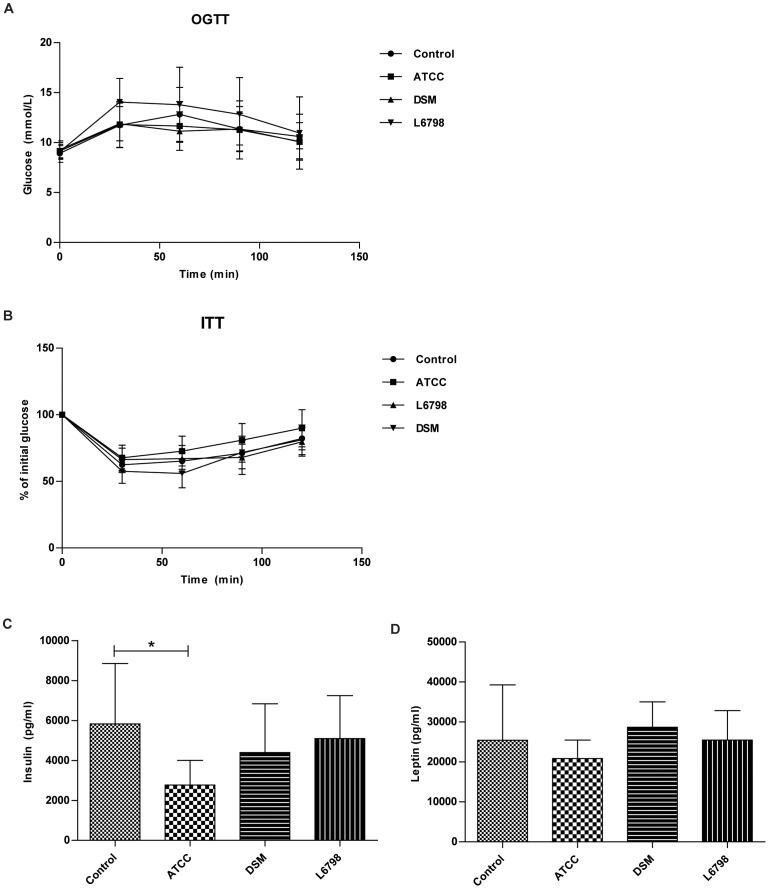
No effects of *L. reuteri* treatment on glucose and insulin tolerance. Oral glucose (A) and insulin (B) tolerance tests and serum insulin (C) and leptin (D) levels of *Apoe−/−* mice treated with either *L. reuteri* ATCC 4659 (ATCC), *L. reuteri* DSM 1798 (DSM), or *L. reuteri* L6798 (L6978) in the drinking water for 12 weeks. Control mice received no bacterial supplement. Mice were fed a high-fat diet with 0.2% cholesterol. Data shown are mean (SD). one-way ANOVA was used to compare control mice and the ATCC, DSM and L6798 groups.

### Serum Analyses

Cytokine levels (IL-1β, TNF-α, INF-γ, IL-10, IL-12, IL-2, IL-4, IL-5 and mKCa) were measured in serum samples using Mesoscale Multiplex plates and insulin, GLP-1 and leptin were quantified by Mesoscale single-plex and duplex plates, respectively (Mesoscale Discovery, Gaithersburg, USA). Total cholesterol and triglyceride levels were analysed by a colorimetric assay (Infinity, Thermo Fisher Scientific Inc., Middletown, USA).

**Figure 3:**
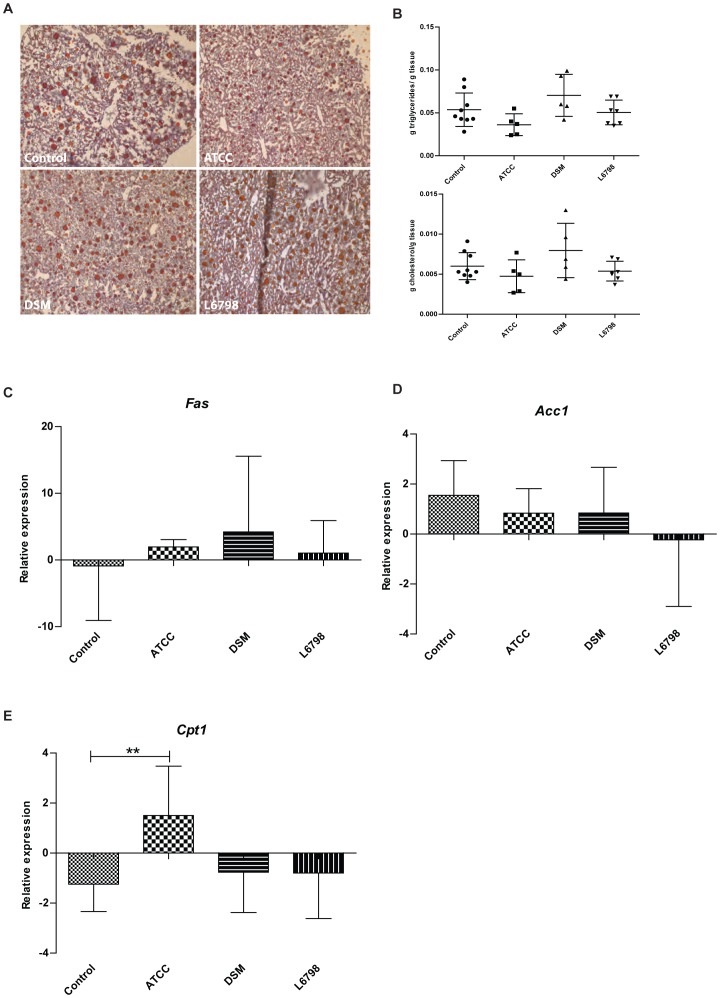
Liver lipids and gene expression of *Apoe−/−* mice treated with *L. reuteri* ATCC 4659, *L. reuteri* DSM 1798 or *L. reuteri* L6798 in the drinking water for 12 weeks. (A) Staining of 8 µm frozen liver sections with Oil-Red-O and haematoxylin/eosin. Stained area vs. total area was calculated to estimate the degree of lipids in the liver. (B) Amount of triglycerides and cholesterol in liver homogenates determined by a colorimetric assay (Infinity, Thermo Fisher Scientific Inc., Middletown, USA). (C–E) Gene expression of *Fas*, *Acc1* and *Cpt1a* in the liver. Expression of the mouse ribosomal protein L32 was used to normalize the expression levels. One-way ANOVA was used to compare control mice and the ATCC, DSM and L6798 groups. Data shown are mean (SD).

### Quantitative Real Time PCR of WAT, Liver and Small Intestine

RNA was extracted from liver and small intestine using the RnEasy Mini kit (Qiagen, Hilden, Germany) and from WAT with RnEasy Lipid Tissue Mini kit (Qiagen, Hilden, Germany). The high capacity cDNA kit was used to synthesize cDNA according to protocol (Applied Biosystems, Carlsbad, USA). SYBR Green based (Biorad) real-time PCR was used to quantify relative expression levels of fatty acid synthase (*Fas*), Acetyl-CoA-carboxylase 1 (*Acc1*) and carnitine palmitotyltransferase 1a (*Cpt1a*) in the liver, angiopoietin-like protein 4 (*Angpt4*; also known as fasting-induced adipose factor) in the small intestine and *Emr1,* which encodes the *F4/80* macrophage marker, in WAT. Expression of the mouse ribosomal protein L32 was used to normalize the expression levels. Primer sequences are provided as Table 1.

**Table 2:** Serum cytokines.

Cytokine	Controln = 10	ATCCn = 10	DSMn = 9	L6798n = 10
**IFN-g**	33 (37)	43 (38)	12 (15)	22 (27)
**IL-10**	382 (464)	349 (238)	140 (97)	150 (160)
**IL-12**	2078 (213)	2123 (376)	2298 (409)	2199 (950)
**IL-1b**	52 (71)	101 (97)	16 (24)	36 (70)
**IL-2**	81 (117)	105 (123)	21 (27)	42 (82)
**IL-4**	44 (58)	76 (75)	21 (21)	29 (37)
**IL-5**	69 (72)	153 (130)	30 (36)	50 (60)
**TNF-a**	45 (58)	59 (59)	39 (51)	56 (72)
**mKCa**	221 (110)	207 (74)	158 (52)	184 (102)

*Apoe−/−* mice were treated with either *L. reuteri* ATCC 4659, *L. reuteri* DSM 1798 or *L. reuteri* L6798 in the drinking water for 12 weeks. Control mice received no bacterial supplement. Mice were fed a high-fat diet with 0.2% cholesterol. Data shown are mean (SD).

### Liver Steatosis

Approximately 50 mg of liver tissue was homogenized in PBS and total cholesterol and TG levels were analysed by a colorimetric assay (Infinity, Thermo Fisher Scientific Inc., Middletown, USA) [13]. In addition, 8 µm frozen sections were cut using a cryostat and stained with Oil-Red-O. Stained area vs. total area was calculated to estimate the degree of lipids in the liver.

**Figure 4:**
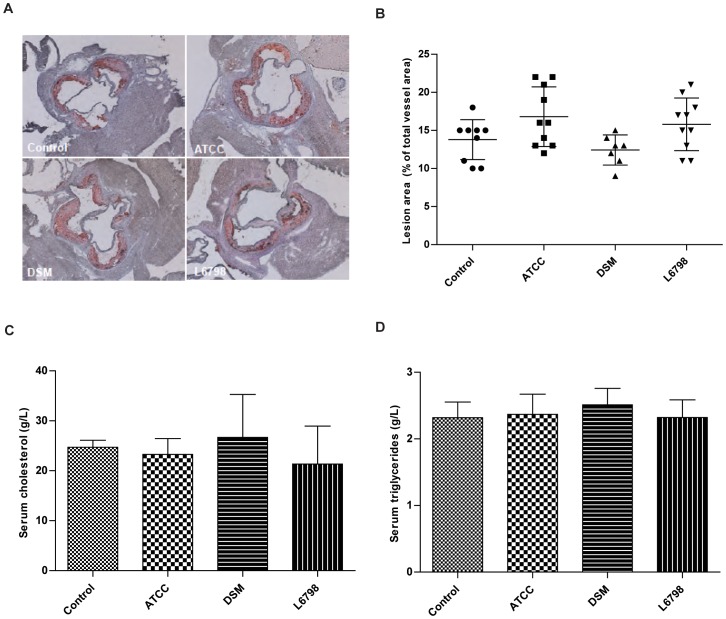
*L. reuteri* treatment does not affect atherosclerosis development in Apoe mice. (A) Atherosclerosis assessment of aortic root region in *Apoe−/−* mice treated with either *L. reuteri* ATCC 4659, *L. reuteri* DSM 1798 or *L. reuteri* L6798 in the drinking water for 12 weeks. (B) Lesion area was designated as stained area/total vessel area and 3 sections/mouse were assessed. (C–D) Serum cholesterol and triglycerides levels. One-way ANOVA was used to compare control mice and the ATCC, DSM and L6798 groups. Data shown are mean (SD).

### ITT and OGTT

Mice were fasted for 4 hours, and injected intraperitoneally with insulin (0.55 U/kg, Novolin-R, Novo Nordisk, Copenhagen, Denmark) or gavaged orally with glucose (3 g/kg b. wt), after which blood glucose levels were measured at 0, 30, 60, 90 and 120 min using tail vein blood (Hemocue, Ängelholm, Sweden).

### Atherosclerosis Assessment

Frozen, 8 µm thick aortic root sections were cut using a cryostat and stained with Oil-Red-O. Lesion area was designated as stained area/total vessel area. Immunohistochemistry was performed on frozen sections with antibodies directed against macrophages (F4/80; 1∶200) and T-cells (CD3; 1∶100) (Abcam, Cambridge, USA) along with the Immpress Mouse adsorbed Peroxidase Kit (Vector Laboratories, Burlingame, USA) according to the manufacturer’s protocol. Sections were counterstained with haematoxylin and three sections/mouse were assessed.

### Statistics

1-way ANOVA and repeated measures ANOVA with Dunnett’s post-hoc test (Graphpad Prism 5, v. 5.02) were used to analyse differences between the Control group and the ATCC, DSM and L6798 groups. Results are mean and standard deviation, unless otherwise noted.

## Results

### Diet-induced Obesity and Insulin Resistance

*Apoe*-deficient mice on a C57BL/6J genetic background develop atherosclerosis and other signs of the metabolic syndrome when fed Western diet supplemented with cholesterol. To investigate whether *L. reuteri* strains modulated metabolic syndrome, especially obesity, glucose metabolism and atherosclerosis, the mice received either (1) a human adapted pro-inflammatory strain which lacks bile salt hydrolase activity (*L. reuteri* DSM 17938), (2) a human adapted anti-inflammatory strain which displays bile salt hydrolase activity (*L. reuteri* ATCC PTA 4659) or (3) a mouse adapted anti-inflammatory strain (*L. reuteri* 6798).

We noted that the ATCC group gained significantly less weight than the control group, whereas the L6798 group gained significantly more (*P*<0.0001 Control vs ATCC, *P*<0.001 Control vs L6798, repeated measures ANOVA, Figure 1A). This corresponded with epididymal WAT weights, as well as liver weights (Figure 1B–C). Importantly, all mice had the same starting weight (25.4±1.8) and consumed the same amount of food (data not shown), thus suggesting strain specific effects. *Angptl4* expression in the small intestine is negatively regulated by the gut microbiota and is involved in fat deposition by inhibiting lipoprotein lipase (LPL) activity [14]. Our analysis revealed that the altered adiposity could not be attributed to regulation of ileal *Angptl4* expression (data not shown), suggesting a different mechanism in contrast to F19 [8].

Increased adiposity is associated with insulin resistance [15], but surprisingly, the ATCC mice exhibited no effects on oral glucose or insulin tolerance compared with the control group (Figure 2A–B). However, the leaner ATCC treated group had reduced fasting serum insulin levels, but similar leptin levels (Figure 2C–D).

### Liver Steatosis

After high-fat feeding the mice developed steatosis, a condition characterized by fat accumulation in the liver, which in humans, is closely linked to insulin resistance and the metabolic syndrome. Both OrO-staining of liver sections and quantification of lipids in liver homogenates showed a trend towards lower fat content of the ATCC livers as compared with the control mice (*P* = 0.16 (OrO staining), *P* = 0.10 (Triglycerides) and *P* = 0.18 (Cholesterol) (Figure 3A–B).

To elucidate whether the ATCC strain affected steatosis by reducing lipogenesis or increasing β-oxidation we analysed the expression of *Fas, Acc1* and *Cpt1a*. We did not observe any regulation of expression of lipogenic enzymes, but we found a significantly higher expression of *Cpt1a* in the ATCC group, suggesting increased β-oxidation in this group of mice (Figure 3C–E).

### Inflammation

The bacterial strains selected in this study possess anti-inflammatory or pro-inflammatory properties [16]. Since obesity is associated with increased inflammation, we investigated whether *L. reuteri* modulated the inflammatory tone by measuring cytokines in the serum (Table 2). In addition, we analysed the expression of the macrophage marker F4/80 in WAT to investigate whether macrophage infiltration was affected by the *L. reuteri* (data not shown). No significant differences regarding these inflammation parameters were found between the bacterial groups and the control group.

### Atherosclerosis

Obesity and insulin resistance are risk factors associated with atherosclerosis and in hypercholesterolemic *Apoe−/−* mice, the aortic root region of the heart is prone to developing large atherosclerotic lesions due to high turbulence and stress on the tissue. No treatment had any effect on atherosclerosis development or on blood cholesterol and triglyceride levels (Figure 4A–D). Furthermore, we did not observe any differences in macrophage or T-cell numbers in plaques (data not shown).

## Discussion

The present study was designed to investigate whether three different *L. reuteri* strains with diverse characteristics could modify diet-induced obesity, glucose metabolism or atherosclerosis in the *Apoe−/−* mouse model. One of the strains, ATCC, partly prevented diet-induced obesity. Since no differences in food intake was observed, we excluded bacterial influence on appetite and instead continued to investigate three mechanisms that might explain the decreased adiposity: 1) decreased inflammation – which has been shown previously to play a role in adiposity in mice [15] –2) increased expression of *Angptl4* and 3) altered liver lipid metabolism. Our results indicate that the ATCC strain predominantly affected *Cpt1a* expression in the liver, which might provide a novel mechanism for how the gut microbiota could affect adiposity. Interestingly, mice given the ATCC strain in their drinking water also displayed lower blood insulin levels and tended to have lower fat content of the liver. Previous literature show discrepancies regarding the gut microbial composition in obese and lean individuals and in some studies, lactobacilli have been enriched in obese patients [17]. However, causality of this phenomenon has yet to be shown and the present study suggests strain-specific effects of lactobacilli on body weight and adiposity.

Fatty acid oxidation has been suggested as a target for the metabolic syndrome [18]. The process of β-oxidation in mitochondria involves shuttling of long chain fatty acids released from adipose tissue across the mitochondrial membrane, a process regulated by the enzymes *Cpt1a* and *Acc2* [18]. Both inhibitors and activators of *Cpt1a* have been proposed to combat hyperglycemia and the metabolic syndrome. *Cpt1a* activity is increased in diabetic patients, as insulin negatively regulates *Cpt1a* [18]. However, inhibitors improving hyperglycemia have severe side effects, such as intracellular lipid accumulation, liver steatosis, insulin resistance and myocardial hypertrophy. Instead, stimulation of *Cpt1a* or interventions in the *Acc* system have had beneficial effects on insulin resistance and adiposity without apparent side effects [18]. Similarly, in the present study, the ATCC strain induced expression of *Cpt1a* in the liver, might provide an alternate mechanism to combat insulin resistance and obesity in a hypercholesterolemic setting.

We also elucidated whether manipulation of the gut microbiota could affect atherosclerosis in *Apoe/−* mice, which has been observed in a few previous studies. For instance, by feeding *Apoe−/−* mice prebiotics for 16 weeks, atherosclerotic lesions were reduced by 35% [19]. In a study in rabbits, the probiotic bacterium *Enterococcus faecium* was able to improve the lipid profile but not atherosclerosis development [20]. The probiotic bacterium *Lactobacillus plantarum* 299 v was able to affect hallmarks of cardiovascular disease in smokers, i.e. reduce systolic blood pressure, leptin and fibrinogen levels [21]. However, none of the strains in the present study were able to reduce lesion size or plaque stability. This might be explained by the fact that the bacteria did not lower blood cholesterol or triglyceride levels, or that the inflammatory status of the mice was not altered by the bacterial treatment.

In summary, the ATCC protected against development of diet-induced obesity, lowered blood insulin levels and appeared to affect liver steatosis in hypercholesterolemic mice on a high-fat diet. In addition, we suggest a previously unknown mechanism for how a probiotic bacterium could influence liver lipid metabolism and whole-body adiposity: *via* increased expression of *Cpt1a*. Bacterial strains of the same species showed different effects on adiposity and insulin sensitivity, illustrating the complexity of host-bacterial cross-talk and the importance of investigating specific bacterial strains.
